# Supplementary material for: Defensin-Rich Platelets Drive Pro-Tumorigenic Programs in Pancreatic Adenocarcinoma
Source: Int J Mol Sci. 2025 Nov 10;26(22):10898. doi: 10.3390/ijms262210898 (PMC12652293; doi:10.3390/ijms262210898)
Supplement: Supplementary file 1 [file ijms-26-10898-s001.zip › Supplementary Figures.pdf]

Defensin-Rich Platelets Drive Pro-Tumorigenic Programs in Pancreatic Adenocarcinoma

Jonathan Gonzalez-Ruiz[1,2], Miryam Sarmiento-Casas[2] and Ivan Bahena-Ocampo[1], Magali Espinosa[2]Gisela Ceballos-Cancino[2], Karla Vázquez-Santillan[2], Vilma Maldonado[4], Jorge Melendez-Zajgla[2,*]

Supplementary Figures

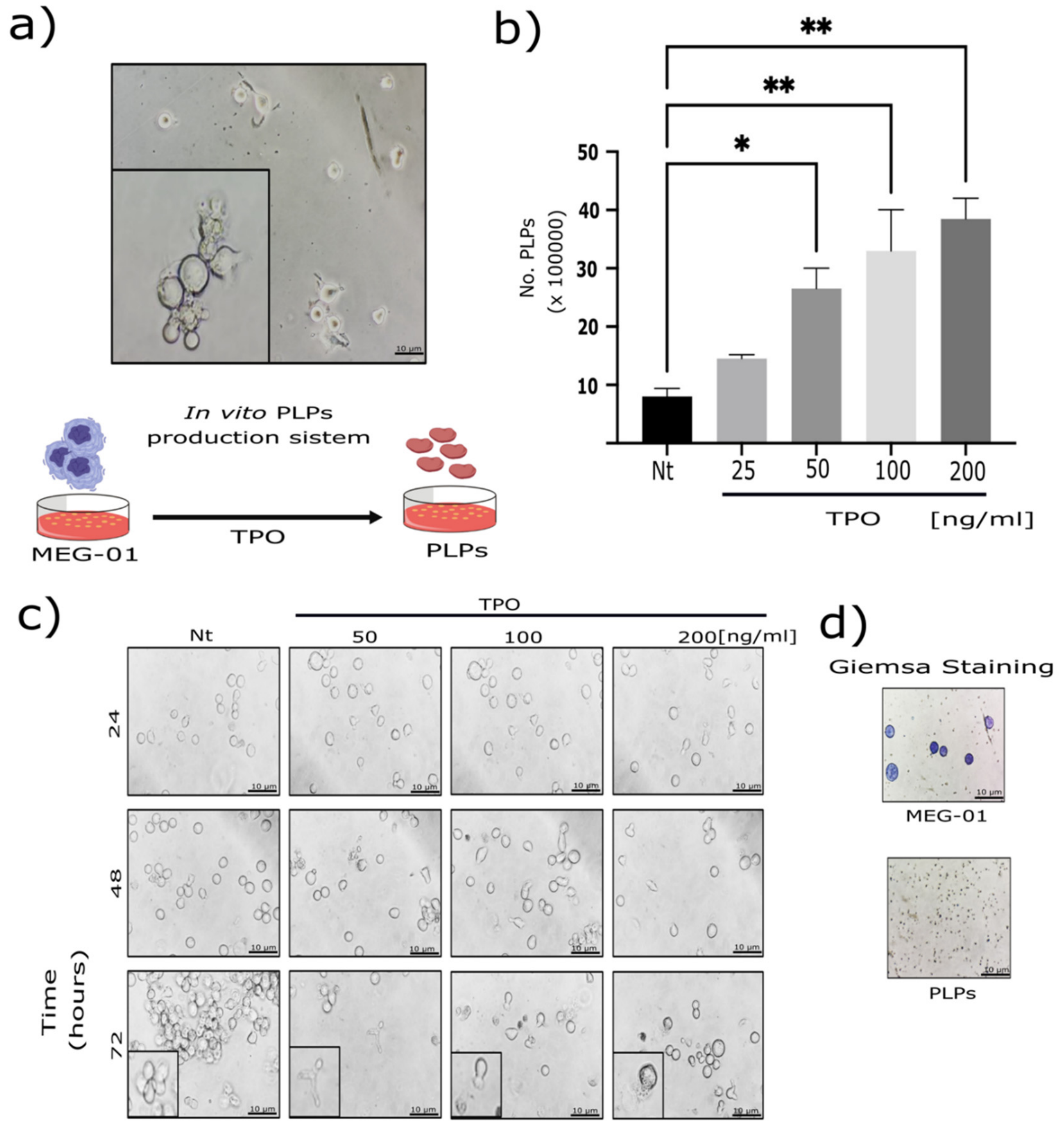

Supplementary Figure S1. Optimization of platelet-like platelet (PLP) generation from MEG-01 cells using thrombopoietin (TPO). To determine the optimal conditions for platelet-like particle (PLP) production, MEG-01 cells were stimulated with increasing concentrations of recombinant

human thrombopoietin (TPO) for 72 hours. **(a)** Schematic representation of the experimental workflow for TPO-induced PLP generation. **(b)** Quantification of PLP yield at different TPO concentrations, showing that treatment with 200 ng/mL TPO for 72 hours resulted in the highest particle production. **(c)** Morphological confirmation of PLP formation by Giemsa staining, illustrating enhanced particle release at the optimal TPO concentration. Scale bar = 10 μm. **(d)** Summary of quantitative data, expressed as mean ± S.E.M. from three independent experiments, each performed in triplicate. Statistical analysis was performed using one-way ANOVA followed by Tukey's post-hoc test. Significance threshold: $^*p \leq 0.05$, $^{**}p \leq 0.01$ compared with lower TPO concentrations. Abbreviations: PLP, platelet-like particle; TPO, thrombopoietin; S.E.M., standard error of the mean.

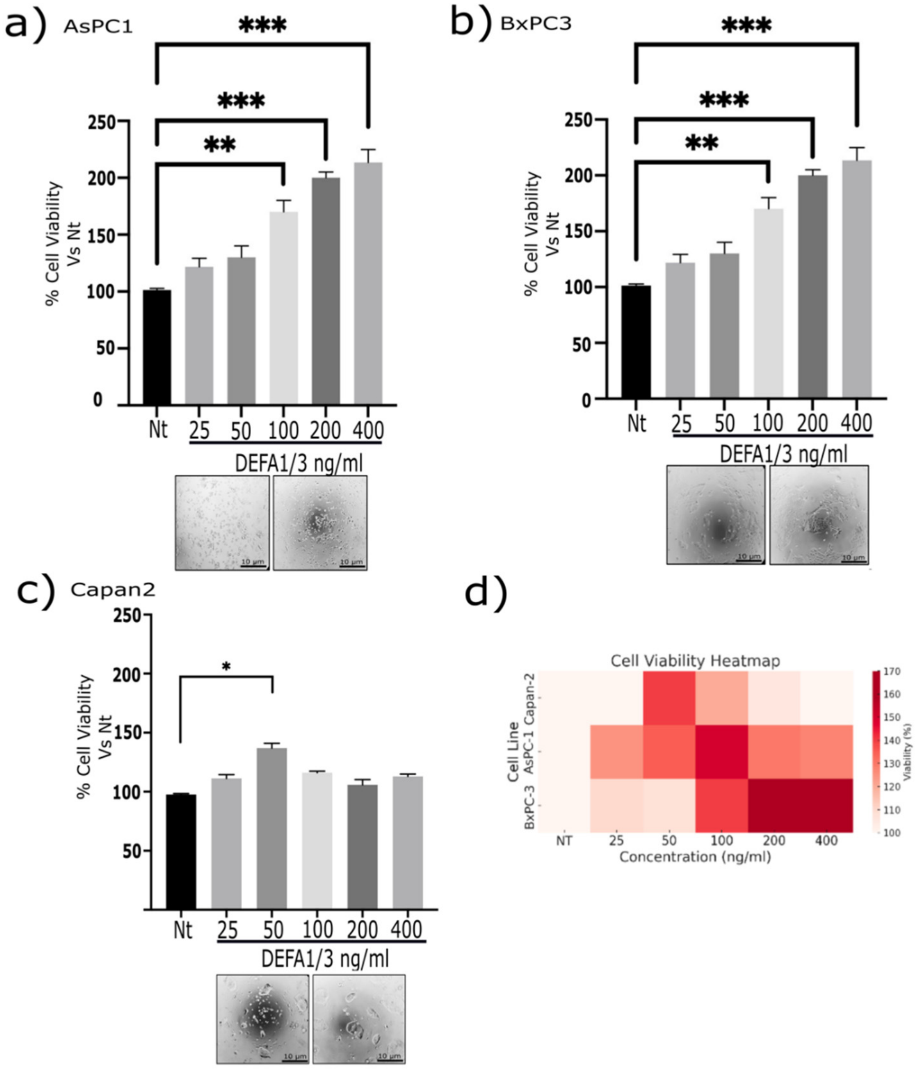

Supplementary Figure S2. To evaluate the effect of recombinant human DEFA1/3 on tumor cell viability, AsPC-1, BxPC-3, and Capan-2 pancreatic cancer cell lines were treated with increasing concentrations of DEFA1/3 (25–400 ng/mL) for 72 hours. **(a–c) Cell viability assays** were performed for each cell line using a colorimetric method based on metabolic activity. All tested

cell lines exhibited a dose-dependent increase in viability in response to DEFA1/3 compared with the non-treated control (Nt) group. **(d) Heatmap representation** summarizing relative viability across concentrations and cell lines, illustrating the progressive increase in cell survival as DEFA1/3 levels rise. Data are presented as mean ± S.E.M. from at least three independent biological replicates, each conducted in triplicate. Statistical analysis was performed using one-way ANOVA followed by Tukey's post-hoc test. Significance threshold: **p* ≤ 0.05, **p ≤ 0.01, ****p* ≤ 0.01 vs. Nt. Abbreviations: DEFA1/3, defensin alpha 1/3; Nt, non-treated; S.E.M., standard error of the mean.

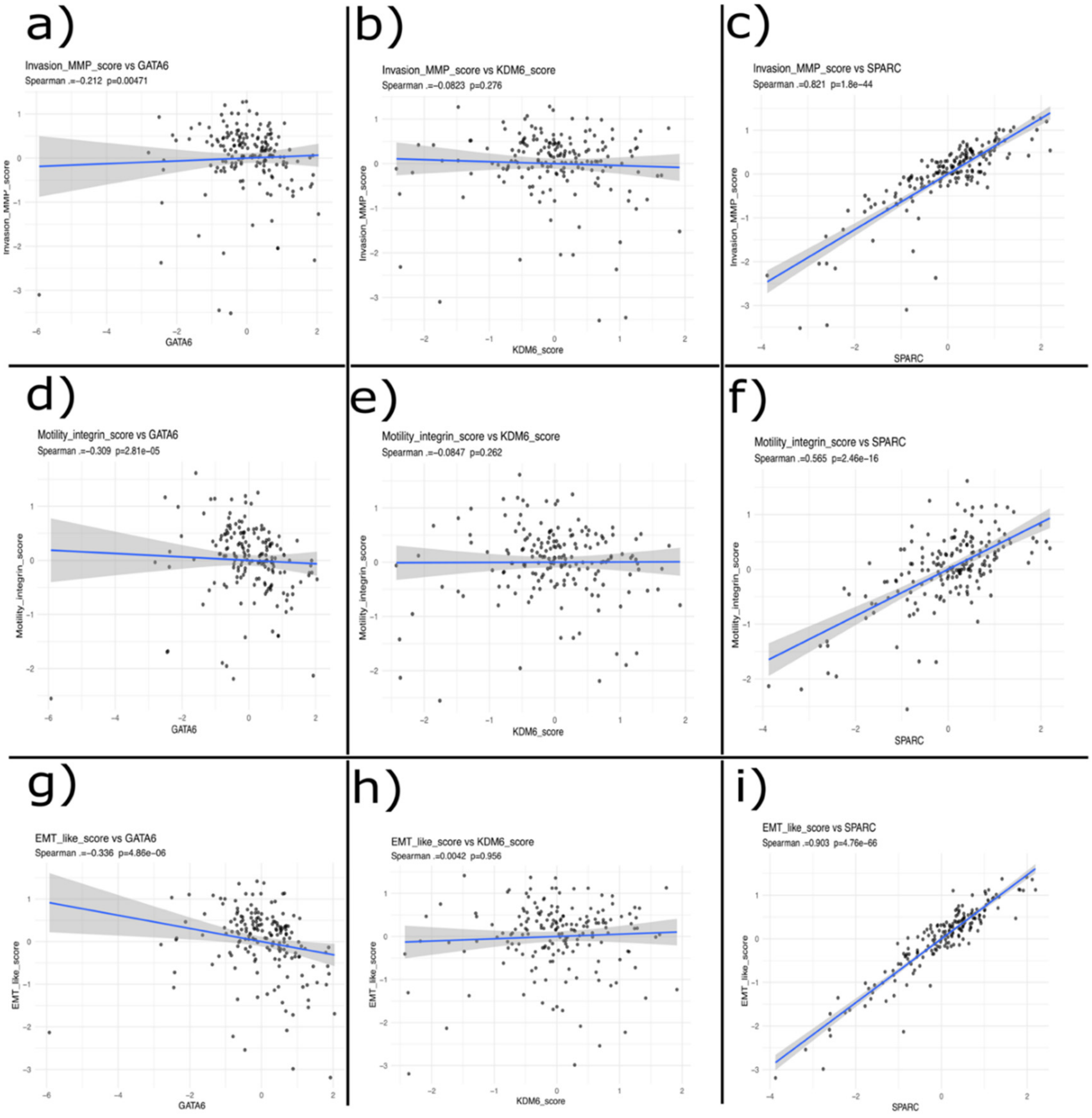

Supplementary Figure S3. Correlation of invasion-, motility-, and EMT-related transcriptional programs with *GATA6*, *KDM6*, and *SPARC* expression in TCGA-PAAD. Spearman correlation analysis between transcriptional scores derived from ssGSEA and the expression of *GATA6*, *KDM6A*, and *SPARC* in TCGA pancreatic adenocarcinoma samples.**(a–c)** Invasion_MMP score

versus GATA6 (rho = –0.212, p = 0.0471) **(a),** *KDM6A* (rho = –0.083, p = 0.276) **(b),** and *SPARC* (rho = 0.821, p = 1.9×10^{-44}) **(c). (d–f)** Motility_integrin score versus GATA6 (rho = –0.306, p = 2.81×10^{-5}) **(d),** *KDM6A* (rho = –0.0847, p = 0.262) **(e),** and *SPARC* (rho = 0.565, p = 2.46×10^{-16}) **(f).** (g–i) EMT_like score versus GATA6 (rho = –0.336, p = 4.86×10^{-6}) (g), *KDM6A* (rho = 0.0042, p = 0.956) **(h),** and *SPARC* (rho = 0.803, p = 4.76×10^{-85}) **(i).** *SPARC* showed strong positive correlations across invasion, motility, and EMT-like programs, whereas *GATA6* correlated negatively with motility and EMT signatures. Abbreviations: EMT, epithelial–mesenchymal transition; ssGSEA, single-sample Gene Set Enrichment Analysis; TCGA-PAAD, The Cancer Genome Atlas–Pancreatic Adenocarcinoma; PDAC, pancreatic ductal adenocarcinoma.
