# Supplementary figures and images for: Defensin-Rich Platelets Drive Pro-Tumorigenic Programs in Pancreatic Adenocarcinoma
Source: Int J Mol Sci. 2025 Nov 10;26(22):10898. doi: 10.3390/ijms262210898 (PMC12652293; doi:10.3390/ijms262210898)

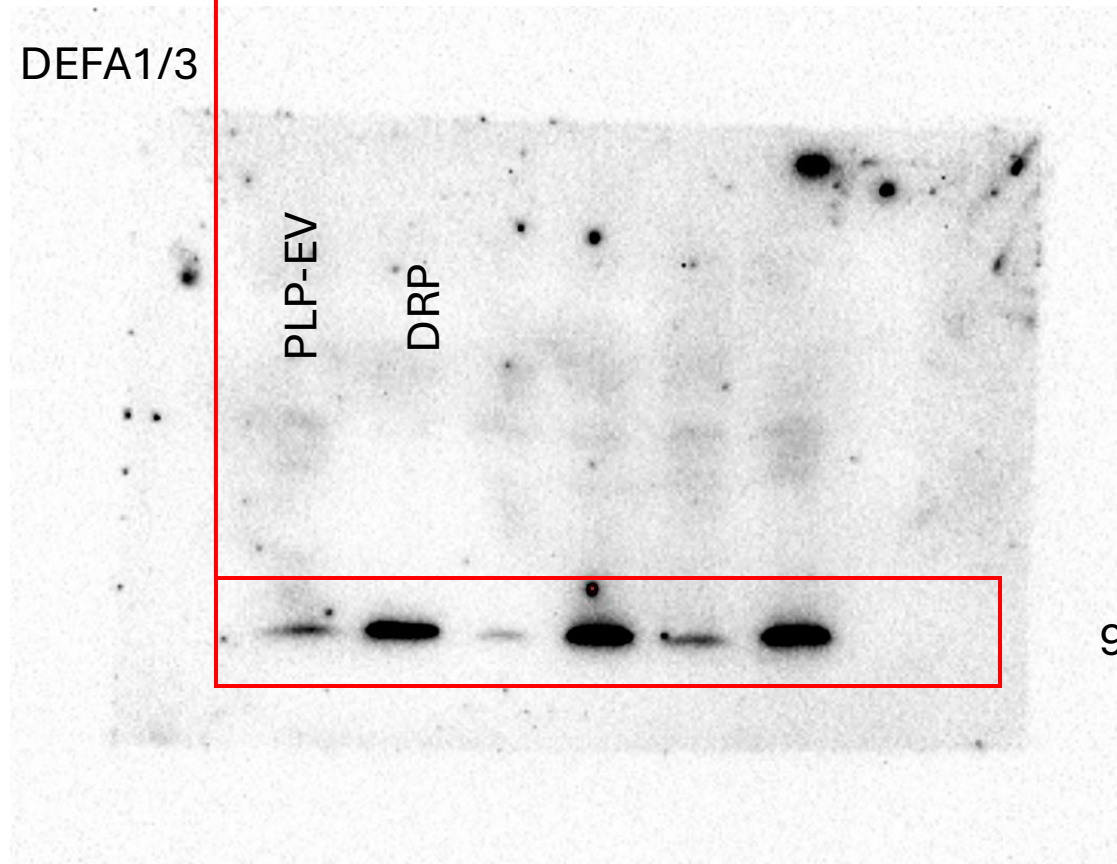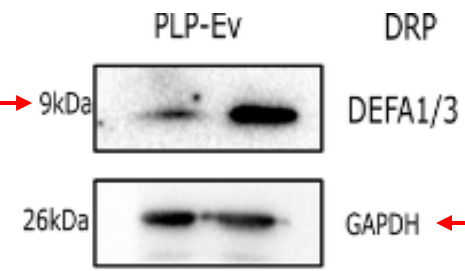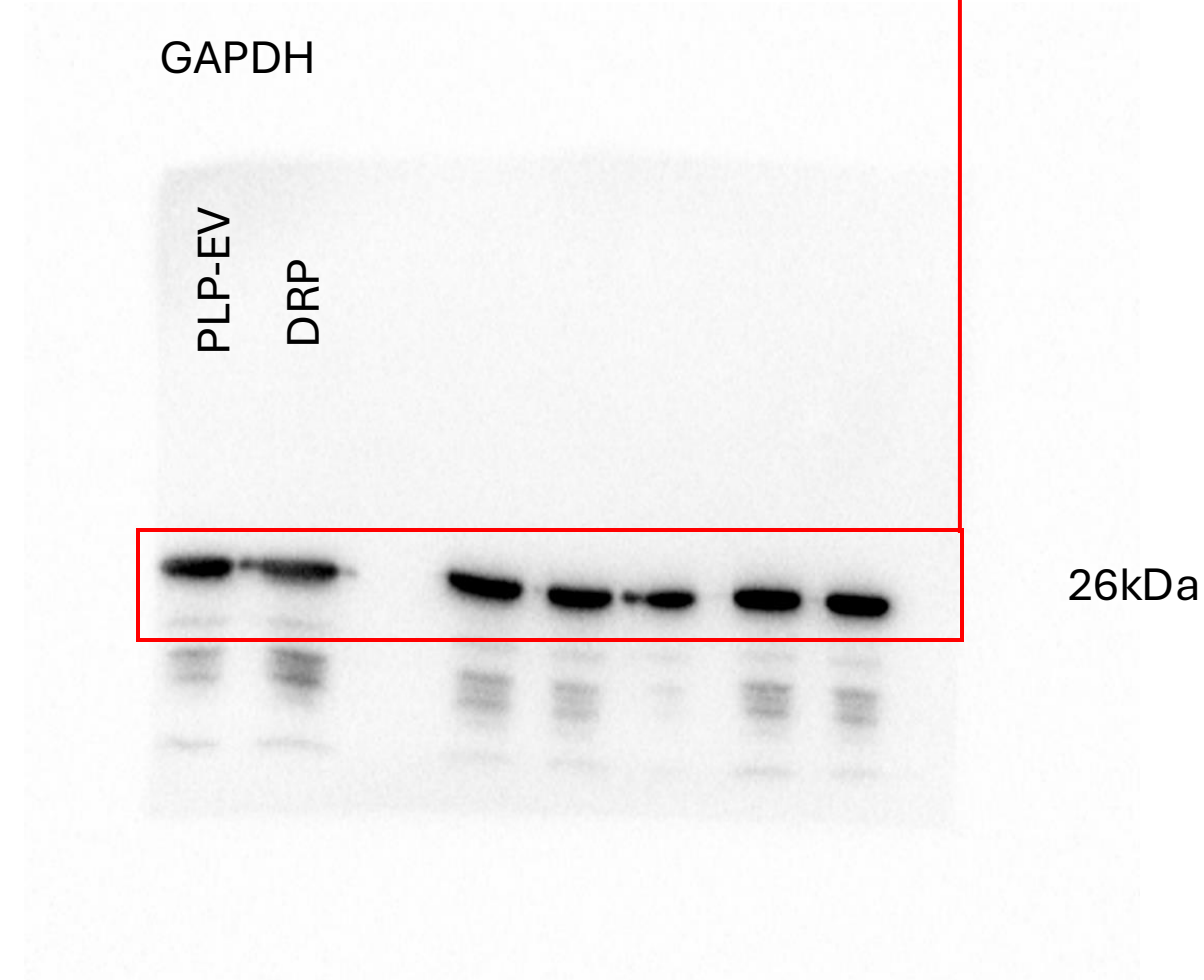

Figure2 e: n= 3 Protein content

Supplement: Supplementary file 1 [file ijms-26-10898-s001.zip › File S1 - Western Blot - DRP_GAPDH.pdf]
